# Type I and II interferons, transcription factors and major histocompatibility complexes were enhanced by knocking down the PRRSV-induced transforming growth factor beta in monocytes co-cultured with peripheral blood lymphocytes

**DOI:** 10.3389/fimmu.2024.1308330

**Published:** 2024-03-06

**Authors:** Dante Fabros, Wasin Charerntantanakul

**Affiliations:** Program of Biotechnology, Faculty of Science, Maejo University, Chiang Mai, Thailand

**Keywords:** porcine reproductive and respiratory syndrome virus, transforming growth factor beta, antisense, innate immunity, type I and II interferons

## Abstract

The innate and adaptive immune responses elicited by porcine reproductive and respiratory syndrome virus (PRRSV) infection are known to be poor. This study investigates the impact of PRRSV-induced transforming growth factor beta 1 (TGFβ1) on the expressions of type I and II interferons (IFNs), transcription factors, major histocompatibility complexes (MHC), anti-inflammatory and pro-inflammatory cytokines in PRRSV-infected co-cultures of monocytes and peripheral blood lymphocytes (PBL). Phosphorothioate-modified antisense oligodeoxynucleotide (AS ODN) specific to the AUG region of porcine TGFβ1 mRNA was synthesized and successfully knocked down TGFβ1 mRNA expression and protein translation. Monocytes transfected with TGFβAS1 ODN, then simultaneously co-cultured with PBL and inoculated with either classical PRRSV-2 (cPRRSV-2) or highly pathogenic PRRSV-2 (HP-PRRSV-2) showed a significant reduction in TGFβ1 mRNA expression and a significant increase in the mRNA expressions of IFNα, IFNγ, MHC-I, MHC-II, signal transducer and activator of transcription 1 (STAT1), and STAT2. Additionally, transfection of TGFβAS1 ODN in the monocyte and PBL co-culture inoculated with cPRRSV-2 significantly increased the mRNA expression of interleukin-12p40 (IL-12p40). PRRSV-2 RNA copy numbers were significantly reduced in monocytes and PBL co-culture transfected with TGFβAS1 ODN compared to the untransfected control. The yields of PRRSV-2 RNA copy numbers in PRRSV-2-inoculated monocytes and PBL co-culture were sustained and reduced by porcine TGFβ1 (rTGFβ1) and recombinant porcine IFNα (rIFNα), respectively. These findings highlight the strategy employed by PRRSV to suppress the innate immune response through the induction of TGFβ expression. The inclusion of TGFβ as a parameter for future PRRSV vaccine and vaccine adjuvant candidates is recommended.

## 1 Introduction

Porcine reproductive and respiratory syndrome virus (PRRSV) is causing global economic loss to the swine industry (1). PRRSV causes chronic respiratory symptoms in pigs of all ages, reproductive failure, abortion, fetal death, and congenital illnesses in pregnant sows (2). It belongs to the genus *Porarterivirus*, family *Arteriviridae*, order *Nidovirales.* PRRSV is divided into two distinct species i.e., *Betaarterivirus suid* 1 (previously known as PRRSV-1) and *Betaarterivirus suid* 2 (previously known as PRRSV-2) (3, 4). Both PRRSV species share up to 60% nucleotide sequence homology and comprise classical PRRSV (cPRRSV) strains and highly pathogenic PRRSV (HP-PRRSV) strains (5).

PRRSV is an enveloped positive single-stranded RNA virus that primarily infects and highly restricts porcine myeloid antigen presenting cells (APCs) i.e., monocytes, macrophages, and dendritic cells (6, 7). Its genome is approximately 15 kb in size, consisting of 10 open reading frames. PRRSV’s structural protein i.e. nucleocapsid (N) (8), and glycoprotein 5 (9) suppress the host’s antiviral interferon (IFN) response by interfering TRIM22 and TRIM25 mediated RIG-1 ubiquitination, downregulating NF-κB and p38 MAPK (10), inhibiting the phosphorylation and nuclear translocation of interferon regulatory factor 3 (IRF3) (11), signal transducer and activator of transcription 2 (STAT2) expression and STAT1 translocation (12) Non-structural protein 1α (nsp1α) (13, 14), nsp1β (15), nsp2 (16), nsp4 (17), nsp5 (18), and nsp11 (19) mediate downregulation of type I IFN-regulated genes, and IFN-stimulated genes (ISGs) by degrading CREB-binding protein (13, 14), IRF-3 and NF-κB (15–17), degrading STAT3 (18) and inhibiting ISGF3 targeting IRF9 (19). On the other hand, upregulation of interleukin-10 (IL-10) and STAT3 by PRRSV’s proteins N (10), nsp1 (20), and GP5 (21) was observed in APCs to skew the antiviral response of the host.

Moreover, PRRSV is thought to be able to infect professional antigen presenting cells, impairing their normal antigen presentation ability by inducing apoptosis, down-regulating the expression of IFNα, major histocompatibility complex-I (MHC-I), MHC-II, CD11b/c and CD14, upregulating the expression of IL-10, and inducing minimal Th1 cytokine secretion (22–24). PRRSV upregulated the frequency of regulatory T-cells (Tregs) (25). The virus could stimulate IL-10 production with the associated generation of Tregs (10). PRRSV-infected monocyte-derived dendritic cells (MoDCs) drastically upregulated transcriptional forkhead pox P3 (FoxP3) for Tregs generation. It also suggested that IL-10 and Tregs could be related to weakened IFNγ production and altered development of protective T-cell response (6, 26).

PRRSV enhances IL-10 expression (27, 28). IL-10 upregulation, in conjunction with limited expressions of interferon-regulated genes, contributes to the downregulation of pro-inflammatory innate immune defense mechanisms e.g., antiviral and phagocytic activities, antigen presentation, pro-inflammatory cytokines and immune-related marker expressions in infected myeloid APCs. The weak and delayed inductions of adaptive cytotoxic T cell (29) and T helper 1 (Th1) cell responses, as well as the promotion of regulatory Tregs differentiation, further facilitate the survival of PRRSV and the development of clinical manifestations (30–32).

Transforming growth factor beta (TGFβ) has three isoforms in mammals, with TGFβ1 being the most abundant isoform and responsible for a wide range of specific responses (33). In pigs, the impact of PRRSV-induced TGFβ1 overexpression on immune protection against PRRSV has not been investigated to date. TGFβ is up-regulated by PRRSV in infected myeloid APCs (34), co-cultivated peripheral blood mononuclear cells (PBMCs) (27), lungs and lymphoid tissues of pigs (35). According to the previous study, TGFβ can enhance the viability of PRRSV-infected cells, thereby contributing to increased PRRSV survival (36). Limited existing reports on the immunoregulatory activities of TGFβ in pigs are available. In porcine monocyte-derived macrophages (MDMs), TGFβ has been reported to down-regulate the expressions of CD14, IL-6, MHCII, and tumor necrosis factor alpha (TNFα) (37). In mice, TGFβ has been found to down-regulate CD14 expression in lipopolysaccharide (LPS)-stimulated macrophages, leading to the suppression of the MyD88-dependent signaling pathway (38, 39). Moreover, TGFβ has been shown to suppress MHCII, IL-12p40, and CD40 expressions in murine macrophages (38), Th1 cell differentiation, Th1-mediated inflammatory response, and the expressions of IFNγ, IL-2, and IL-4 (40) as well as the activation of macrophages, dendritic cells (DCs), and natural killer cells (38). In contrast, TGFβ promotes Tregs differentiation through the up-regulation of Smad3 and Foxp3 expressions (41).

This study aims to investigate the effects of PRRSV-induced TGFβ on the expressions of immune-related genes in PRRSV-inoculated monocytes and PBL co-culture. Monocytes were transfected with phosphorothioate-modified antisense (AS) oligodeoxynucleotide (ODN) targeting the AUG region of porcine TGFβ1 mRNA to knockdown its expression. The present study reveals that TGFβ exerts a regulatory role by down-regulating gene expressions of type I and II IFNs, MHCs, and STATs in PRRSV-inoculated monocytes and PBL co-culture. These findings provide valuable insights into potential targets and strategies for augmenting the innate and cell-mediated immune (CMI) responses to PRRSV vaccines and vaccine adjuvants.

## 2 Materials and methods

### 2.1 Virus

Thai cPRRSV-2 (01NP1) (42) and HP-PRRSV-2 (10PL1) (43) were propagated in MARC-145 cells grown in minimum essential medium (MEM; Caisson Laboratories, Smithfield, UT), 10% heat-inactivated fetal bovine serum (FBS; Capricorn Scientific GmbH, Germany) streptomycin (100 µg/ml), penicillin (100 IU/ml), and amphotericin B (250 ng/ml) (all from Gibco, NY) to 10^6^ TCID50/ml. Supernatants from uninoculated MARC-145 cell lysate were served as mock Ag.

### 2.2 Pigs

Eight 24-week-old PRRSV-seronegative crossbred pigs (large white/landrace x duroc) were the source of peripheral blood mononuclear cells (PBMCs) to obtain monocytes and PBLs. They were housed at the swine research farm, faculty of animal science and technology, Maejo University.

### 2.3 Optimization of real-time PCR conditions

PBMC isolation was conducted as described previously (44). Briefly, PBMCs were isolated from whole blood by density gradient centrifugation using Histopaque^®^-1077 (Sigma, St. Louis, MO). Contaminating red blood cells were lysed by cold lysis buffer (1 mM EDTA, 0.156 M ammonium chloride and 10 mM sodium bicarbonate). PBMCs were resuspended in roswell park memorial institute-1640 (RPMI-1640) with L-glutamine (Caisson Laboratories), 10% heat-inactivated FBS, penicillin (100 IU/ml), streptomycin (100 μg/ml) and amphotericin B (250 ng/ml) to 10^6^ cells. In this study, RPMI-1640 added with FBS and antibiotic/antimycotic i.e., penicillin, streptomycin and amphotericin B was named as RPMI^++^. Two hundred μl of PBMC suspension (2 x 10^6^ cells) was seeded onto 96-well flat-bottom plates (Nunc, Denmark), and received 50 µl of either Concanavalin A (ConA) (10 μg/ml final conc.) (TargetMol, MA) or phorbol 12-myristate 13-acetate (7 ng/ml final conc.) (TargetMol, MA) plus ionomycin (430 ng/ml, final conc.) (APExBIO, TX) (PMAi). The final concentrations of ConA and PMAi used in this study were the least concentrations that could induce detectable mRNA expressions of all immune-related genes of interest (45, 46). PBMCs were stimulated for 18 h (37°C, humidified 5% CO_2_) prior to RNA isolation.

Total RNA was isolated using PureLink™ RNA mini kit (Invitrogen, USA). The quality and quantity of RNA were evaluated by the OneDrop TOUCH Pro/Lite Micro-Volume Spectrophotometer (Biometrics Technologies Inc. USA). All RNA samples had A260/230 and A260/280 between 1.8-2.2 and 2.0-2.2, respectively. The integrity of RNA was determined by denaturing agarose gel electrophoresis followed by ethidium bromide staining. cDNA was carried out using the ReverTra Ace^®^ qPCR RT Kit (Toyobo, Japan). The reaction used 1,000 ng of pooled total RNA as a template, and a mixture of random hexamers and oligo-dT as primers. cDNAs were stored at -20°C until real-time PCR.

Real-time PCR was performed on a QIAquant 96 thermal cycler. A total reaction volume of 20 μl comprised 2 μl serial 5-fold dilutions of pooled cDNA template (starting at 1 µg), 10 μl SYBR^®^ Green real time PCR master mix (Toyobo, Japan), and varying concentrations (200-500 nM) of primer pairs for FOXP3, GATA3 (gata binding protein 3), IFNα, IFNγ, IL-2, IL-4, IL-6, IL-10, IL-12p40, IL-17, MHC-I, MHC-II, RORγT (retinoic acid receptor-related orphan nuclear receptor gamma-t), RPL32 (ribosomal protein L32), STAT1, STAT2, STAT6, T-bet (t-box expressed in T-cells), TGFβ1, TNFα, YWHAZ (tyrosine 3-monooxygenase/tryptophan 5-monooxygenase activation protein, zeta) (**Additional File 1**). All reactions were set up in duplicate. The PCR condition was 95°C (10 min); and 40 cycles of 94°C (15s), designated annealing temperature of 50-60°C (30s), and 72°C (30s), followed by melting curve analysis and agarose gel electrophoresis of PCR products. Band intensities were documented under ultraviolet light (GelMax™ Imager, UVP, CA). A nuclease-free water was included as no template control in every run.

### 2.4 Antisense oligodeoxynucleotides

Phosphorothioate-modified AS-ODNs (TGFβAS1; 5’-AGCCCCGAAGGCGGCATG-3’ and Scramble control (Scr); 5’-GCCGCTTGCTCGCGCCTA-3’) were synthesized by Integrated DNA Technologies (IDT, Singapore).

### 2.5 Transfection of monocytes with AS ODNs

The preparation of monocytes was conducted as previously described (44). Briefly, 100 µl of PBMCs (1 x 10^6^ cells) were seeded onto a 96-well flat bottom plate and incubated for 4 h (37°C, humidified 5% CO_2_). Non-adherent cells were removed, and adherent monocytes were washed twice with 150 µl of pre-warmed (37°C) Opti-MEM^®^ I.

**Figure 1:**
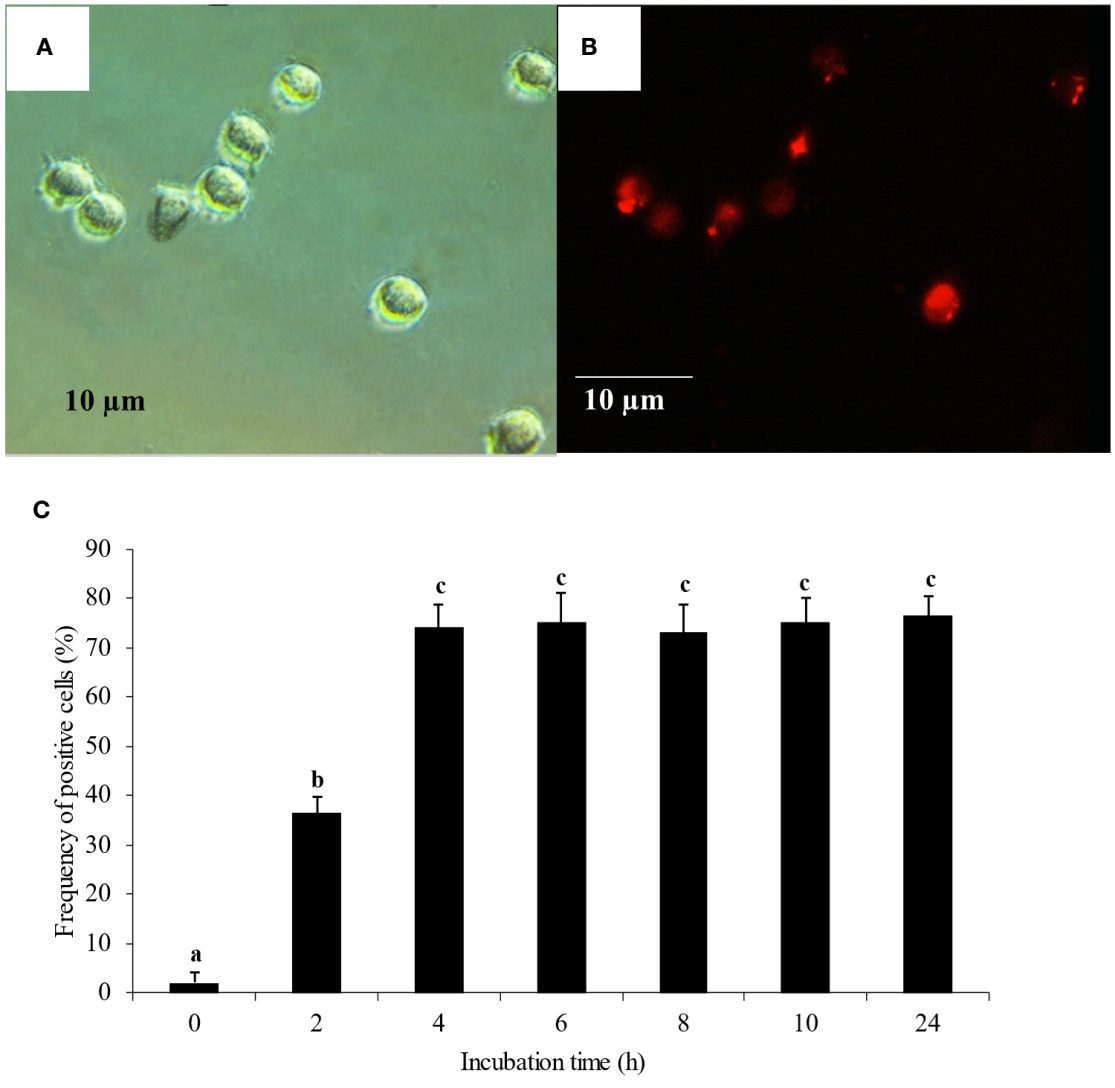
Monocyte transfection and uptake **(A)** Monocytes under bright field microscopy. **(B)** Monocyte uptake of fluorescent-labeled siRNA under immunofluorescent microscopy. **(C)** Monocyte uptake of fluorescent-labeled siRNA complexed with 1.5% of transfection reagent. The uptake was observed every 2 h for 10 h and finally at 24 h. Mean differences of percentages of fluoresced cells among groups were tested by one-way ANOVA, followed by Tukey HSD test. Different letters indicate significant differences. P<0.05 was set as a statistically significant level.

Transfection was carried out following the guidelines of Lipofectamine™ RNAiMax (Invitrogen, CA) with the recommended small interfering RNA (siRNA, BLOCK-iT™ Alexa Fluor^®^ Red Fluorescent control, Invitrogen). Mixtures of 0.75, 1.5 and 3% Lipofectamine™ RNAiMax in Opti-MEM^®^ I (v/v) and 2 µM siRNA suspended in Opti-MEM^®^ I were incubated at room temperature (RT) for 30 min. Twenty µl of each mixture was added to the wells containing monocytes and gently mixed by rocking for 5 min. Monocyte uptake of fluorescent-labeled siRNA (**Figures 1A, B**) was observed under an immunofluorescent microscope (Nikon Eclipse Ti, Japan) every 2 h for 10 h and finally at 24 h. Frequencies of immunofluorescent-positive cells were identified using automatic measurement for cell counting (NIS-elements software ver. 3.22, Nikon, Japan). Cell viability was determined by trypan blue staining. Optimal concentration of transfection reagent and optimal transfection period were determined.

### 2.6 TGFβ1 knockdown in monocytes then co-cultured with peripheral blood lymphocytes prior to the evaluation of immune-related gene expressions

Monocytes were prepared as described above while PBLs were prepared as previously described (44). Monocytes were washed with 100 µl pre-warmed (37°C) Opti-MEM^®^ I and transfected with 40 µl TGFβAS1 mixture for 4 h (37°C, humidified 5% CO_2_). The transfection condition was based on the results of optimization of transfection reagent concentration and transfection period (**Figure 1C**, **Additional File 2**). The TGFβAS1 mixture was removed, and adherent TGFβAS1-transfected monocytes were washed. Then, 200 µl of PBL (2 x 10^6^ cells) in RPMI^++^ from the same animal were added to the TGFβAS1-transfected monocyte for co-culture. The ratio of PBL added to monocytes resembled the ratio of lymphocytes to monocytes in the peripheral blood of pigs of the same age, approximately 10:1 (47). Finally, 50 µl of inducer, either ConA (10 μg/ml final conc.) or PMAi (7 and 430 ng/ml final conc.) was added to the wells and incubated for an additional 12 h (37°C, humidified 5% CO_2_).

Cell culture supernatants were collected for subsequent determination of TGFβ1 protein levels by ELISA (Porcine TGF Beta 1 PicoKine™ ELISA kit, Boster Biological Technology, Pleasanton, CA). Monocytes and PBL co-cultures were harvested prior to RNA isolation and generation of cDNA as described above. Unspecific knockdown of immune-related genes, i.e., FoxP3, GATA3, IFNα, IFNγ, IL-2, IL-4, IL-6, IL-10, IL-12p40, IL-17, MHC-I, MHC-II, RORγT, STAT1, STAT2, STAT6, T-bet, TGFβ1 and TNFα was determined by real-time PCR. Positive, negative, Scr, and transfection media controls were included.

For the determination of mRNA expression levels of TGFβ1 and other immune-related genes (**Additional File 1**), 200 ng of total RNA was used as template for cDNA synthesis as described above. The threshold cycles (C_T_) of all genes were used for the calculation of gene expression by the 2^(-ΔΔC_T_) method. The expressions of TGFβ1 and other immune-related genes were normalized to the geometric average of RPL32 (ribosomal protein L32) and YWHAZ (tyrosine 3-monooxygenase/tryptophan 5-monooxygenase activation protein, zeta) and calibrated to that in the negative control. The expression levels of all immune-related genes were transformed into log2 scale.

### 2.7 Evaluation of TGFβ1 knockdown effects on immune-related gene expressions in monocytes co-cultured with PBL and inoculated with cPRRSV-2 and HP-PRRSV-2

Monocytes were transfected with 2 µM TGFβAS1 for 4 h. The TGFβAS1 mixture was removed, and monocytes were washed. TGFβAS1-transfected monocytes were inoculated with 100 µl of either cPRRSV-2 or HP-PRRSV-2 equivalent to a multiplicity of infection (m.o.i.) of 1. At the time of PRRSV-2 inoculation, each monocyte culture simultaneously received 100 µl of PBL (1 x 10^6^ cells) from the same animal. The cultures were incubated for 48 h (37°C, humidified 5% CO_2_), then received 50 µl of inducers. The cultures were incubated further for 12 h (37°C, humidified 5% CO_2_). Cells were then harvested prior to RNA isolation. Cell culture supernatants were collected for the determination of TGFβ1 protein levels by ELISA. Expressions of immune-related genes were determined every 12 h by real-time PCR. Controls included cells receiving mock Ag plus inducers (mock control); cells receiving PRRSV-2 and inducers (PRRSV-2-inoculated control); and cells treated with transfection media alone (without TGFβAS1), inoculated with PRRSV-2, and stimulated with inducers (PRRSV-inoculated/transfection media control). Untreated cells receiving culture media in the presence or absence of inducers served as positive and negative controls, respectively. Cell viability was determined at the end of the transfection period, PRRSV-2 inoculation, and inducer stimulation using trypan blue.

### 2.8 Evaluation of TGFβ1 knockdown effects on PRRSV RNA yields in monocytes and PBL co-cultures inoculated with cPRRSV-2 and HP-PRRSV-2

Monocytes were transfected with TGFβAS1 (2 µM) in transfection media as described above. Subsequently, transfection media were removed and replaced with 100 µl of either cPRRSV-2 or HP-PRRSV-2 (equivalent to m.o.i. of 1). Monocyte cultures were incubated for 1 h (37°C, humidified 5% CO_2_), then the supernatants were discarded. The monocytes were then washed twice with 150 µl pre-warmed (37°C) RPMI^++^ and received 200 µl PBL (2 x 10^6^ cells) prior to the addition of 50 µl inducers. The cultures were incubated further for 12 h (37°C, humidified 5% CO_2_) prior to RNA isolation. Cell culture supernatants (150 µl) were collected for quantification of PRRSV-2 ORF7 RNA by real-time PCR (48). Controls included cells receiving PRRSV-2 and inducers (PRRSV-2-inoculated control); cells transfected with Scr, inoculated with PRRSV-2, and stimulated with inducers (PRRSV-2-inoculated/Scr control); and cells treated with transfection media alone, inoculated with PRRSV-2, and stimulated with inducers (PRRSV-2-inoculated/transfection media control). Cells receiving mock Ag plus inducers served as uninoculated controls.

PRRSV-2 RNA was isolated and contaminating DNA was eliminated using PureLink™ RNA mini kit (Invitrogen, USA). The quality and quantity of RNA were determined by OneDrop TOUCH Pro/Lite Micro-Volume Spectrophotometer (Biometrics Technologies Inc. USA). Reverse transcription (using the ReverTra Ace^®^ qPCR RT Kit, Toyobo, Japan) and real-time PCR were conducted as described previously (48). In brief, a total reaction volume of 20 µl, consisting of 2 µl cDNA, 10 µl SYBR^®^ Green PCR master mix (Toyobo), and 400 nM each of primers ORF7 149F and ORF7 346R was set up in duplicate. The PCR condition was 95°C (15 min); and 35 cycles of 95°C (15 s), 53°C (30 s), and 72°C (30 s). The C_T_ was collected and compared with the standard curve of C_T_ generated from 10^1^-10^8^ copy numbers of recombinant PRRSV-2 ORF7 plasmids. Melting curve analysis and agarose gel electrophoresis were performed to verify a single product. Nuclease-free water was included as no template control in every run.

### 2.9 Evaluation of TGFβ1 and IFNα protein effects on PRRSV RNA yields in monocytes and PBL co-culture inoculated with cPRRSV-2 and HP-PRRSV-2

#### 2.9.1 Effect of porcine IFNα and TGFβ1 concentrations

Monocytes were treated with 100 µl of either recombinant porcine TGFβ1 (rTGFβ1; Raybiotech, GA) or recombinant porcine IFNα (rIFNα; Raybiotech, GA) resuspended in pre-warmed RPMI^++^ at 10, 1, and 0.1 ng/ml final. The cultures were incubated for 24 h (37°C, humidified 5% CO_2_), then received 100 µl of either cPRRSV-2 or HP-PRRSV-2 (equivalent to m.o.i. of 1). The cultures were incubated for 1 h (37°C, humidified 5% CO_2_), then washed and added with 200 µl PBL (2 x 10^6^ cells) and 50 µl inducers. The cultures were incubated further for 12 h (37°C, humidified 5% CO_2_) prior to RNA isolation. Cell culture supernatants (150 µl) were collected for quantification of PRRSV-2 ORF7 RNA by real-time PCR. Controls included cells receiving PRRSV-2 and inducers (PRRSV-2-inoculated control), and cells receiving mock Ag plus inducers (uninoculated control). Cell viability was determined at the end of culture periods using trypan blue.

#### 2.9.2 Effects of TGFβ1 and IFNα on PRRSV RNA yields in monocytes and PBL co-culture inoculated with cPRRSV-2 and HP-PRRSV-2

Monocytes were treated with 100 µl of rTGFβ1 (10 ng/ml final) resuspended in pre-warmed RPMI^++^. The monocyte cultures were incubated for 24 h (37°C, humidified 5% CO_2_) prior to receiving 50 µl of rIFNα (10 ng/ml final). The cultures were further incubated for 24 h (37°C, humidified 5% CO_2_), then received 100 µl of either cPRRSV-2 or HP-PRRSV-2 (equivalent to m.o.i. of 1). The monocyte cultures were incubated for 1 h (37°C, humidified 5% CO_2_), and then the supernatants were removed. Monocytes cultures were washed, added with 200 µl PBL (2 x 10^6^ cells), and received 50 µl inducers. The cultures were incubated further for 12 h (37°C, humidified 5% CO_2_) prior to RNA isolation. Cell culture supernatants (150 µl) were collected for quantification of PRRSV-2 ORF7 RNA by real-time PCR. Controls included cells receiving PRRSV-2 and inducers (PRRSV-2-inoculated control); cells receiving rTGFβ1, PRRSV-2, and inducers (rTGFβ1-treated/PRRSV-2-inoculated control); cells receiving rIFNα, PRRSV-2, and inducers (rIFNα-treated/PRRSV-2-inoculated control); and cells receiving mock Ag plus inducers (uninoculated control). Cell viability was determined at the end of culture periods using trypan blue.

### 2.10 Statistical analysis

Statistical analyses were performed using the SPSS software version 28 (IBM, Armonk, NY). Mean differences of immune-related gene expressions among groups were tested by one-way ANOVA, followed by Tukey HSD test. Mean differences of PRRSV-2 ORF7 copy numbers and immune-related gene expressions among groups at time points were tested by one-way repeated measures ANOVA, followed by Tukey HSD test. P<0.05 was set as a statistically significant level.

## 3 Results

### 3.1 Efficient knockdown of TGFβ1 mRNA expression by TGFβAS1

Tr. media of 1.5% (v/v) complexed with fluorescent-labeled siRNA control and a transfection period of 4 h had yielded the highest transfection efficiency with approximately 74% fluorescent-positive monocytes (**Figure 1C**). These conditions were therefore used for subsequent transfection experiments. Tr. media of 0.75% and 3% were also tested (**Additional File 2**) to confirm the effect of transfection media concentrations, in which 0.75% transfection media yielded the lowest transfection efficiency and 3% Tr. media had no significant difference to 1.5% Tr. media in transfection efficiency.

**Figure 2:**
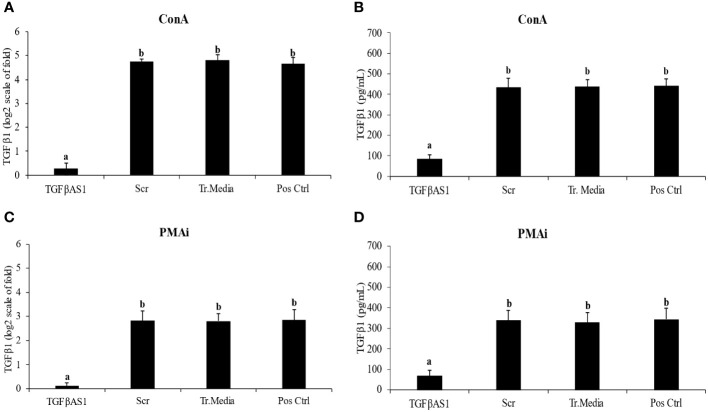
Effect of TGFβ1 antisense (TGFβAS1), and scramble (Scr) phosphorothioate-modified ODNs on expression of TGFβ1 mRNA and protein translation in monocyte and PBL co-culture. Monocytes were transfected with TGFβAS1 or Scr, added with PBL, and then stimulated with either **(A, B)** ConA or **(C, D)** PMAi. Monocytes transfected with transfection media (Tr.media) alone, added with PBL, and finally stimulated with inducers (either ConA or PMAi) served as Tr.media control. Untransfected monocytes, added with PBL, and finally stimulated with inducers served as positive control (Pos Ctrl). Cell supernatant was collected prior to ELISA. Data were normalized to the geometric average of RPL32 and YWHAZ in relative to untransfected/unstimulated monocytes and PBL co-culture. Band intensities indicate the quality of TGFβ1 knockdown (**Additional File 3**). Error bars indicate the standard deviation (SD). Mean differences of TGFβ1 gene expression or protein translation among groups were tested by one-way ANOVA, followed by Tukey HSD test. Different letters above the error bars indicate significant differences. Data are presented in log 2 scale of “fold” according to 2^(-ΔΔC_T_) method.

TGFβ1 mRNA expression (**Figure 2A**) and protein translation (**Figure 2B**) were efficiently downregulated by TGFβAS1 in ConA-stimulated monocytes and PBL co-culture. The same downregulation by TGFβAS1 was observed in PMAi-stimulated monocytes and PBL co-culture (**Figures 2C, D**). TGFβAS1 targeted the AUG region of porcine TGFβ1 mRNA and had no activity-decreasing motifs to ensure the antisense activity. Scr and Tr.media had no significant effect on TGFβ1 mRNA expression when compared to positive control.

### 3.2 Specificity of TGFβAS1 knockdown

Using BLAST, the specificity of TGFβAS1 and Scr was evaluated and analyzed. TGFβAS1 was specific to porcine TGFβ1 mRNA and had no aligned target in any porcine immune-related genes reported in this study or essential genes involved in the porcine immune system. Also, Scr had no aligned target in any of the porcine genes of interest. TGFβAS1 also had no aligned target in any ORFs of PRRSV-2 strains used in this study.

Slightly reduced mRNA expressions of FoxP3 (1.3 ± 0.3 vs 2.0 ± 0.4) and IL-10 (4.0 ± 0.2 vs 4.6 ± 0.3) as compared to positive control were observed in TGFβAS1-transfected monocytes co-cultured with PBL (**Table 1**). On the other hand, slightly increased mRNA expressions of IFNγ (5.5 ± 0.2 vs 4.8 ± 0.2), MHC-I (3.1 ± 0.3 vs 2.4 ± 0.4), and STAT2 (2.4 ± 0.3 vs 1.9 ± 0.4) were demonstrated in TGFβAS1-transfected monocytes then co-cultured with PBL, when compared to positive control. But these changes in mRNA expression levels were not statistically significant. No significant changes in immune-related mRNA expression levels were observed in Scr and Tr. Media controls as compared to positive control.

**Table 1:** Expression levels of immune-related genes in monocytes transfected with either TGFβAS1 or Scr1, or otherwise treated with transfection media (Tr. media) alone prior to simultaneous addition of PBL for co-culture and stimulated with inducers, either ConA or PMAi.

Gene	TGFβAS1	Scr	Tr. Media	Pos Ctrl
FoxP3	1.3 ± 0.3	2.1 ± 0.4	2.1 ± 0.3	2.0 ± 0.4
GATA3	2.0 ± 0.3	2.5 ± 0.2	2.5 ± 0.1	2.4 ± 0.3
IFNα	3.6 ± 0.4	3.4 ± 0.3	3.6 ± 0.2	3.5 ± 0.2
IFNγ	5.5 ± 0.2	5.1 ± 0.4	5.0 ± 0.3	4.8 ± 0.2
IL-2	2.0 ± 0.4	2.1 ± 0.3	2.1 ± 0.2	2.0 ± 0.4
IL-4	2.7 ± 0.4	3.0 ± 0.4	2.7 ± 0.3	2.7 ± 0.4
IL-6	3.5 ± 0.2	3.4 ± 0.2	3.2 ± 0.2	3.1 ± 0.4
IL-10	4.0 ± 0.2	4.4 ± 0.1	4.5 ± 0.1	4.6 ± 0.3
IL-12p40	4.3 ± 0.2	3.9 ± 0.3	3.9 ± 0.3	4.0 ± 0.4
IL-17	2.6 ± 0.4	2.7 ± 0.3	2.9 ± 0.2	2.9 ± 0.2
MHC-I	3.1 ± 0.3	2.8 ± 0.2	2.7 ± 0.3	2.4 ± 0.4
MHC-II	2.3 ± 0.4	2.3 ± 0.3	2.2 ± 0.2	2.3 ± 0.2
RORγT	3.3 ± 0.3	3.7 ± 0.2	3.7 ± 0.2	3.7 ± 0.1
STAT1	2.0 ± 0.3	1.6 ± 0.3	1.6 ± 0.3	1.7 ± 0.3
STAT2	2.4 ± 0.3	2.0 ± 0.2	2.2 ± 0.2	1.9 ± 0.4
STAT6	2.4 ± 0.3	2.3 ± 0.1	2.1 ± 0.2	2.2 ± 0.3
T-bet	3.9 ± 0.3	4.0 ± 0.3	3.6 ± 0.3	3.7 ± 0.2
TNFα	4.5 ± 0.1	4.7 ± 0.2	4.7 ± 0.2	4.4 ± 0.3

Untransfected monocytes co-cultured with PBL and stimulated with either ConA (for IFNα, IFNγ, IL-6, IL-17, IL-12p40, RORγT, Stat1, T-bet, TNFα) or PMAi (for MHC-I, MHC-II, IL-2, IL-4, FoxP3, Stat2, Stat6, GATA3, IL-10) served as positive control (Pos Ctrl). Data were normalized to the geometric average of RPL32 and YWHAZ in relative to untransfected/unstimulated monocytes and PBL co-culture. Data are presented in log 2 scale of “fold” according to 2^(-ΔΔCT) method (Mean ± SD).

### 3.3 TGFβAS1 significantly knockdown TGFβ1 mRNA expression which was up-regulated by cPRRSV-2 and HP-PRRSV-2, and contributed to improve gene expressions of type I and II IFNs, MHCs, transcription factors which were down-regulated by the viruses

**Figure 3:**
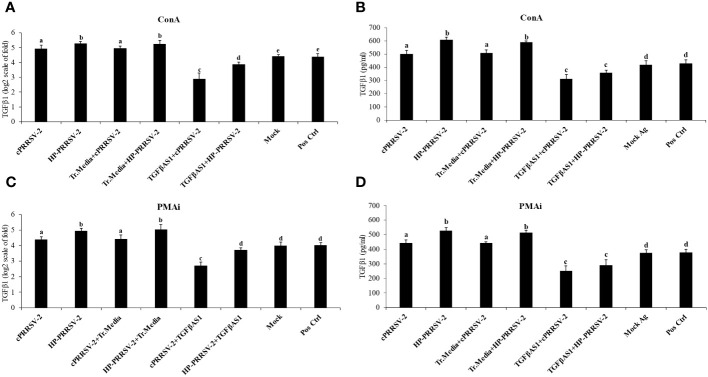
Effect of TGFβAS1 on TGFβ1 mRNA expression and protein translation in PRRSV-2-inoculated PBMCs. Monocytes were transfected with TGFβAS1, then co-cultured with PBL, inoculated with either cPRRSV-2 or HP-PRRSV-2, and stimulated with inducers of either **(A, B)** ConA or **(C, D)** PMAi. Untransfected monocytes, co-cultured with PBL and inoculated with cPRRSV-2 or HP-PRRSV-2, stimulated with inducers, served as the PRRSV-2-inoculated control. Monocytes treated with transfection media (Tr. media), co-cultured with PBL, and inoculated with cPRRSV-2 or HP-PRRSV-2, then stimulated with inducers served as PRRSV-2-inoculated/Tr. media control. Untransfected monocytes, co-cultured with PBL, inoculated with mock Ag, and stimulated with inducers served as mock control. Untreated monocytes then co-cultured with PBL and receiving culture media in the presence or absence of inducers served as positive and negative controls, respectively. **(B, D)** Cell culture supernatants were collected for ELISA. Error bars indicate the SD. Mean differences of TGFβ1 protein translation among groups were tested by one-way ANOVA, followed by Tukey HSD test. Different letters indicate significant differences. P<0.05 was set as a statistically significant level.

Compared to cPRRSV-2-inoculated and HP-PRRSV-2-inoculated monocytes co-cultured with PBL, TGFβAS1+cPRRSV-2-treated and TGFβAS1+HP-PRRSV-2-treated monocytes co-cultured with PBL significantly down-regulated TGFβ1 mRNA expression (**Figures 3A, C**) and protein production (**Figures 3B, D**).

**Figure 4:**
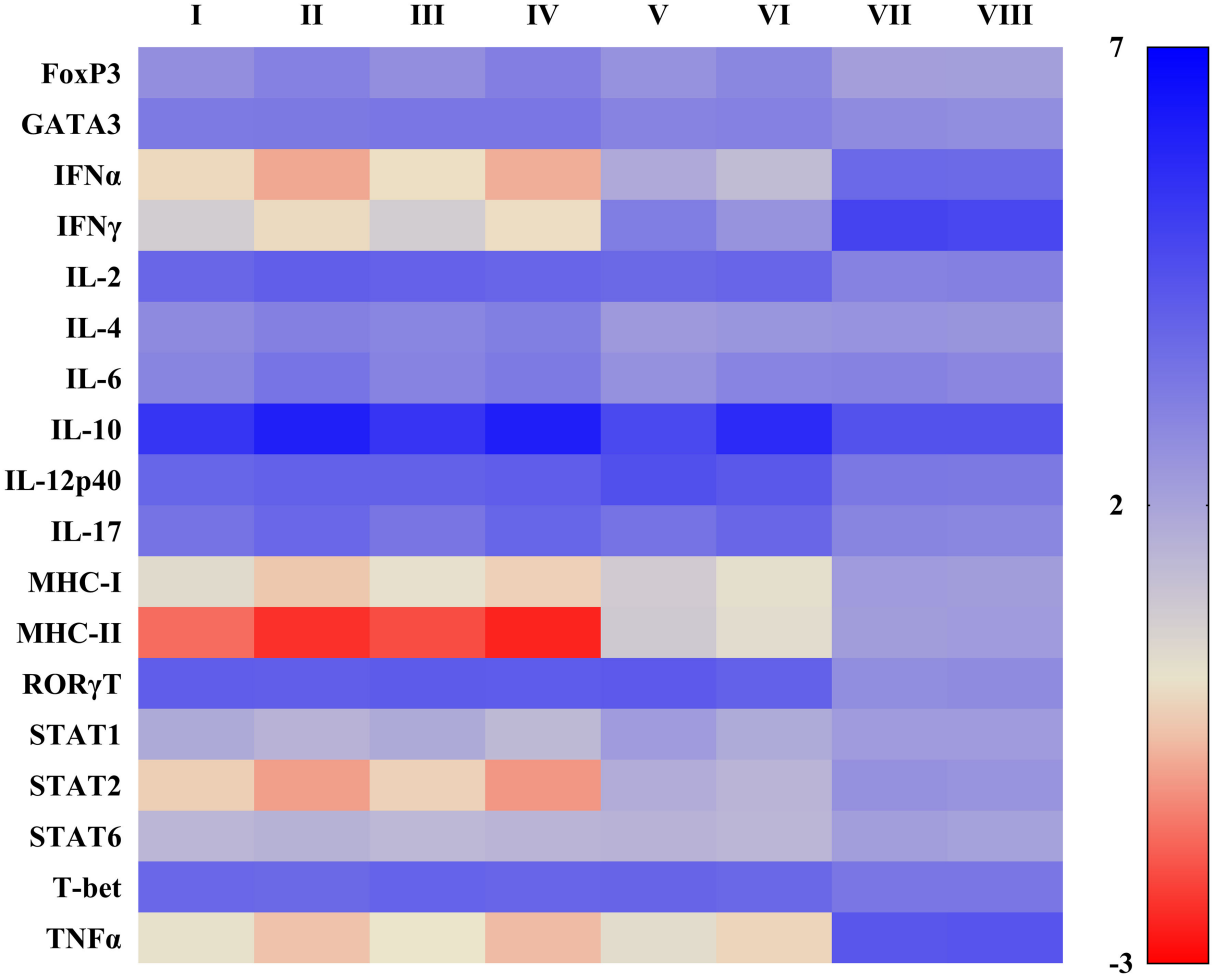
Heat map illustrating effects of TGFβAS1 on immune-related gene expressions in PRRSV-2-inoculated monocytes and PBL co-cultures. Monocytes were transfected with TGFβAS1 prior to the simultaneous addition of PBL and inoculation with either cPRRSV-2 or HP-PRRSV-2, then finally stimulated with either ConA or PMAi. Monocytes transfected with TGFβAS1, co-cultured with PBL, inoculated with either cPRRSV-2 or HP-PRRSV-2, and stimulated with either ConA or PMAi served as the PRRSV-2-inoculated control. Monocytes treated with transfection media (Tr. media), co-cultured with PBL, inoculated with cPRRSV-2 or HP-PRRSV-2, and then stimulated with either ConA or PMAi served as PRRSV-2-inoculated/Tr. media control. Monocytes inoculated with mock Ag, co-cultured with PBL, and stimulated with either ConA or PMAi served as mock control. Untreated monocytes, co-cultured with PBL, stimulated with either ConA (for IFNα, IFNγ, IL-6, IL-17, IL-12p40, RORγT, Stat1, T-bet, TNFα) or PMAi (for MHC-I, MHC-II, IL-2, IL-4, FoxP3, Stat2, Stat6, GATA3, IL-10) served as positive control (Pos Ctrl). I = cPRRSV-2; II = HP-PRRSV-2; III = Tr. media + cPRRSV-2; IV = Tr. media + HP-PRRSV-2; V = TGFβAS1 + cPRRSV-2; VI = TGFβAS1 + HP-PRRSV-2; VII = Mock Ag; VIII = Pos Ctrl. Data were normalized to the geometric average of RPL32 and YWHAZ in relative to untransfected/unstimulated monocytes and PBL co-cultures. Data are presented in log 2 scale of “fold” according to 2^(-ΔΔC_T_) method.

Compared to positive control, cPRRSV-2-inoculated and HP-PRRSV-2-inoculated monocytes co-cultured with PBL significantly upregulated the mRNA expression levels of TGFβ1 (**Figures 3A, C**), FoxP3, GATA3, IL-2, IL-4, IL-10, IL-12p40, IL-17, RORγT, and T-bet (**Figure 4**, **Additional File 4**). HP-PRRSV-2-inoculated monocytes co-cultured with PBL significantly had higher mRNA expression levels of Foxp3, IL-4 and IL-10 than cPRRSV-2-inoculated monocytes co-cultured with PBL. On the other hand, IFNα, IFNγ, MHC-I, MHC-II, STAT1, STAT2, STAT6, and TNFα were significantly downregulated on both cPRRSV-2-inoculated and HP-PRRSV-2-inoculated monocytes co-cultured with PBL as compared to positive control (**Figure 4**, **Additional File 4**). Mock Ag has no significant effect on the expression levels of these immune-related genes as compared to positive control.

Compared to cPRRSV-2-inoculated and HP-PRRSV-2-inoculated monocytes co-cultured with PBL, TGFβAS1+cPRRSV-2-treated and TGFβAS1+HP-PRRSV-2-treated cells significantly down-regulated IL-4 and IL-10 mRNA expression levels (**Figure 4**, **Additional File 4**). On the other hand, significantly increased IFNα, IFNγ, IL-12p40, MHC-I, MHC-II, STAT1, and STAT2 mRNA expression levels in TGFβAS1+cPRRSV-2-treated monocytes co-cultured with PBL were observed as compared to cPRRSV-2-inoculated monocytes co-cultured with PBL. Also, mRNA expression levels of IFNα, IFNγ, MHC-I, MHC-II, and STAT1, STAT2 were significantly increased in TGFβAS1+HP-PRRSV-2-treated monocytes co-cultured with PBL as compared to HP-PRRSV-2-inoculated monocytes and PBL co-culture. Moreover, TNFα expression in TGFβAS1+cPRRSV-2-treated and TGFβAS1+HP-PRRSV-2-treated monocytes co-cultured with PBL had a higher mRNA expression level than cPRRSV-2-inoculated and TGFβAS1+HP-PRRSV-2-treated monocytes co-cultured with PBL, respectively, but showed not statistically significant.

No significant difference was observed with the mRNA expressions of FoxP3, GATA3, IL-2, IL-6, IL-17, and STAT6 between TGFβAS1+cPRRSV-2/HP-PRRSV-2-treated and cPRRSV-2/HP-PRRSV-2-inoculated monocytes and PBL co-culture. Transfection media has no significant effect on the expression levels of these immune-related genes as compared to positive control.

### 3.4 Significantly reduced amounts of cPRRSV-2 and HP-PRRSV-2 RNA yields were influenced by TGFβ1 knockdown

**Figure 5:**
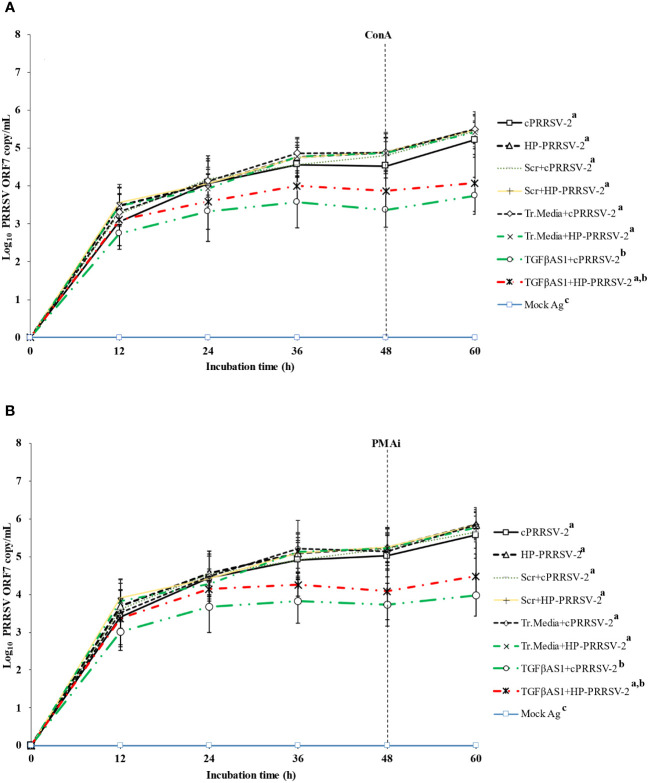
Effect of TGFβ knockdown on PRRSV copy numbers in PRRSV-2-inoculated PBMCs. Monocytes were transfected with TGFβAS1, then inoculated with either cPRRSV-2 or HP-PRRSV-2 and co-cultured with PBL (0 h), and finally stimulated with inducers i.e., **(A)** ConA or **(B)** PMAi (48 h). Monocytes inoculated with cPRRSV-2 or HP-PRRSV-2, then co-cultured with PBL and stimulated with inducer served as the PRRSV-2-inoculated control. Monocytes transfected with Scr, then inoculated with either cPRRSV-2 or HP-PRRSV-2, co-cultured with PBL, and finally stimulated with inducer served as PRRSV-2-inoculated/Scr control. Monocytes treated with transfection media (Tr.media), inoculated with cPRRSV-2 or HP-PRRSV-2, then co-cultured with PBL, and finally stimulated with inducer served as PRRSV-2-inoculated/Tr. media control. Cells receiving mock Ag plus inducer served as uninoculated control. Cell culture supernatants were collected for real-time PCR. The C_T_ values were obtained, and PRRSV-2 ORF7 RNA copy numbers were calculated based on the C_T_ standard curve generated from 10^1^-10^8^ copies of recombinant PRRSV-2 ORF7 plasmids. Data were presented in log 10 scale of copy number/ml. Error bars indicate the SD. Mean differences of PRRSV-2 ORF7 RNA copy numbers among groups at time points were tested by one-way repeated measures ANOVA, followed by Tukey HSD. Different superscript letters indicate significant difference. P<0.05 was set as a statistically significant level.

Compared to cPRRSV-2-inoculated monocytes co-cultured with PBL, monocytes transfected with TGFβAS1 prior to cPRRSV-2 inoculation and PBL co-culture demonstrated significantly lower amount of PRRSV-2 ORF7 RNA copy numbers at 12, 24, 36,48, and 60 h after inoculation (**Figures 5A, B**). Compared to HP-PRRSV-2-inoculated monocytes co-cultured with PBL, monocytes transfected with TGFβAS1 prior to HP-PRRSV-2 inoculation and PBL co-culture demonstrated significantly lower amount of PRRSV-2 ORF7 RNA copy numbers at 12, 24, 36, 48, and 60 h after inoculation (**Figures 5A, B**). Scr and Tr. media had no effect on the alteration of PRRSV-2 ORF7 RNA copy numbers. With mock Ag-treated cells, no PRRSV-2 ORF RNA was detected.

### 3.5 Increased amounts of cPRRSV-2 and HP-PRRSV-2 RNA yields were significantly influenced by TGFβ1

**Figure 6:**
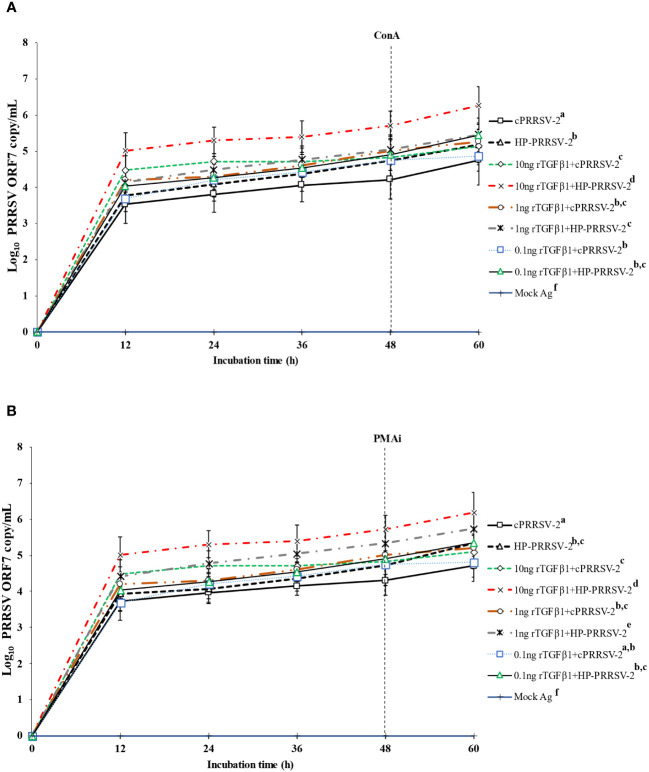
Effects of rTGFβ1 on PRRSV-2 ORF7 RNA copy numbers in PRRSV-2-inoculated monocytes co-cultured with PBL. Monocytes were treated with rTGFβ1 (10, 1 and 0.1 ng/ml final), inoculated with either cPRRSV-2 or HP-PRRSV-2, then co-cultured with PBL (0 h), and finally stimulated with inducers i.e., **(A)** ConA or **(B)** PMAi (48 h). Monocytes inoculated with cPRRSV-2 or HP-PRRSV-2, then co-cultured with PBL and stimulated with inducers served as PRRSV-2-inoculated control. Monocytes receiving mock Ag then co-cultured with PBL plus inducer served as uninoculated control. Cell culture supernatants were collected for real-time PCR. The C_T_ values were obtained and PRRSV-2 ORF7 RNA copy numbers were calculated based on the C_T_ standard curve generated from 10^1^-10^8^ copies of recombinant PRRSV-2 ORF7 plasmids. Data were presented in log 10 scale of copy number/ml. Error bars indicate the SD. Mean differences of PRRSV-2 ORF7 RNA copy numbers among groups at time points were tested by one-way repeated measures ANOVA, followed by Tukey HSD. Different superscript letters indicate significant difference. P<0.05 was set as a statistically significant level.

Compared to cPRRSV-2-inoculated monocytes co-cultured with PBL, rTGFβ1-treated (10 ng/ml final) monocytes inoculated with cPRRSV-2 and then co-cultured with PBL showed significantly higher amount of PRRSV-2 ORF7 RNA copy numbers at 12, 24, 36, 48, and 60 h after inoculation (**Figures 6A, B**). Monocytes treated with rTGFβ1 (1 and 0.1 ng/ml final) prior to cPRRSV-2 inoculation and PBL co-culture had a higher amount of PRRSV-2 ORF7 RNA copy numbers after inoculation.

Compared to HP-PRRSV-2-inoculated monocytes co-cultured with PBL, rTGFβ1-treated (10 ng/ml final) monocytes inoculated with HP-PRRSV-2 and then co-cultured with PBL displayed significantly higher amount of PRRSV-2 ORF7 RNA copy numbers at 12, 24, 36, 48, and 60 h after inoculation (**Figures 6A, B**). Monocytes treated with rTGFβ1 (1 and 0.1 ng/ml final) and then inoculated with HP-PRRSV-2 prior to the co-culture of PBL did not demonstrate a lower amount of PRRSV-2 ORF7 RNA copy numbers after inoculation.

### 3.6 Decreased amounts of cPRRSV-2 and HP-PRRSV-2 RNA yields were significantly influenced by IFNα

**Figure 7:**
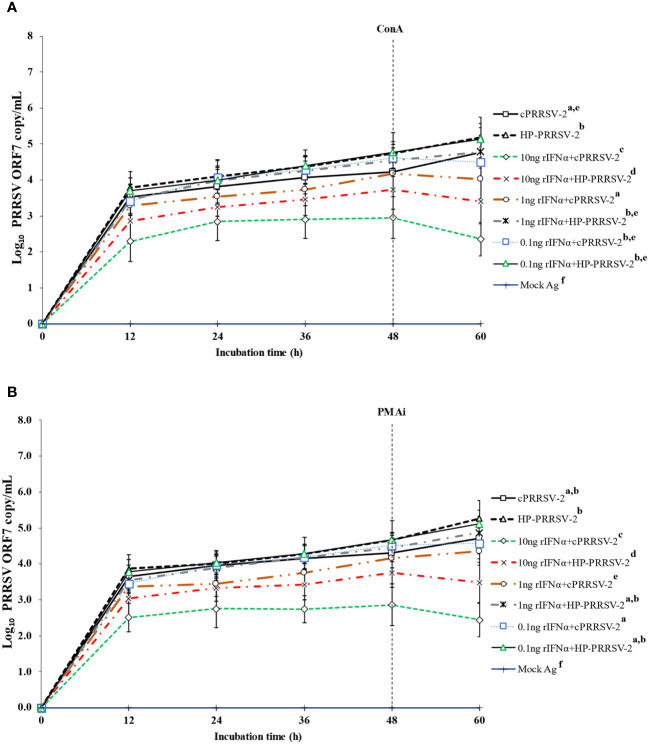
Effects of rIFNα on PRRSV-2 ORF7 RNA copy numbers in PRRSV-2-inoculated monocytes co-cultured with PBL. Monocytes were treated with rIFNα (10, 1 and 0.1 ng/ml final), inoculated with either cPRRSV-2 or HP-PRRSV-2, then co-cultured with PBL (0 h), and finally stimulated with inducers i.e., **(A)** ConA or **(B)** PMAi (48 h). Monocytes inoculated with cPRRSV-2 or HP-PRRSV-2, co-cultured with PBL, and finally stimulated with inducers served as PRRSV-2-inoculated control. Monocytes receiving mock Ag then co-cultured with PBL plus inducer served as uninoculated control. Cell culture supernatants were collected for real-time PCR. The C_T_ values were obtained and PRRSV-2 ORF7 RNA copy numbers were calculated based on the C_T_ standard curve generated from 10^1^-10^8^ copies of recombinant PRRSV-2 ORF7 plasmids. Data were presented in log 10 scale of copy number/ml. Error bars indicate the SD. Mean differences of PRRSV-2 ORF7 RNA copy numbers among groups at time points were tested by one-way repeated measures ANOVA, followed by Tukey HSD. Different superscript letters indicate significant difference. P<0.05 was set as a statistically significant level.

Compared to cPRRSV-2-inoculated monocytes co-cultured with PBL, rIFNα-treated (10 ng/ml final) monocytes inoculated with cPRRSV-2 prior to PBL co-culture demonstrated significantly lower amount of PRRSV-2 ORF7 RNA copy numbers at 12, 24, 36, 48, and 60 h after inoculation (**Figures 7A, B**). Monocytes treated with rIFNα (1 and 0.1 ng/ml final) and then inoculated with cPRRSV-2 prior to PBL co-culture did not demonstrate a lower amount of PRRSV-2 ORF7 RNA copy numbers after inoculation.

Similarly, as compared to HP-PRRSV-2-inoculated monocytes co-cultured with PBL, rIFNα-treated (10 ng/ml final) monocytes inoculated with HP-PRRSV-2 and then co-cultured with PBL demonstrated significantly lower amount of PRRSV-2 ORF7 RNA copy numbers at 12, 24, 36, 48, and 60 h after inoculation (**Figures 7A, B**). Also, monocytes treated with rIFNα (1 and 0.1 ng/ml final) prior to HP-PRRSV-2 inoculation and co-culture with PBL did not demonstrate a lower amount of PRRSV-2 ORF7 RNA copy numbers after inoculation.

### 3.7 Increased amounts of cPRRSV-2 and HP-PRRSV-2 RNA yields were significantly contributed by TGFβ1 which decreased the anti-PRRSV effect of IFNα

**Figure 8:**
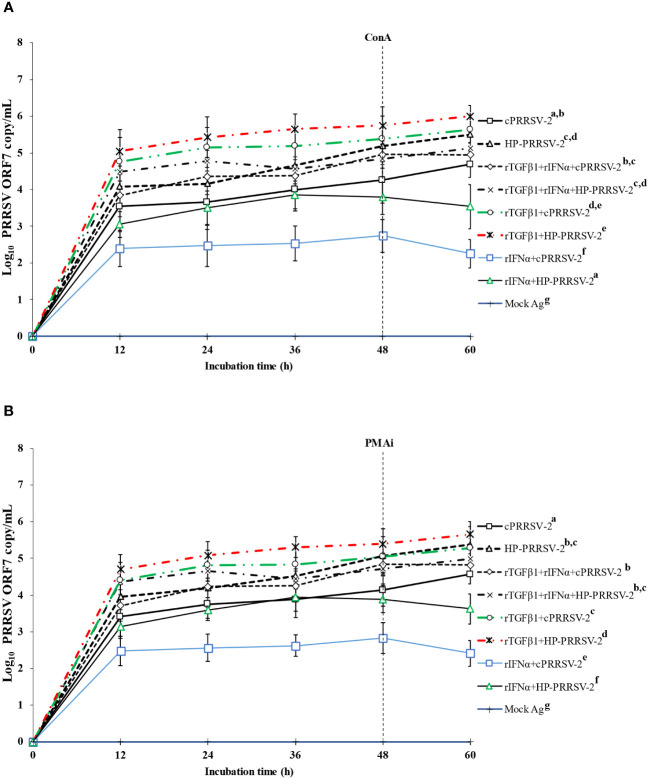
Effects of rTGFβ1 and rIFNα on PRRSV-2 ORF7 RNA copy numbers in PRRSV-2-inoculated monocytes then co-cultured with lymphocytes. Monocytes were treated with rTGFβ1 (10 ng/ml final), followed by rIFNα (10 ng/ml final), then inoculated with either cPRRSV-2 or HP-PRRSV-2, then co-cultured with PBL (0 h), and stimulated with inducers i.e., **(A)** ConA or **(B)** PMAi (48 h). Monocytes inoculated with cPRRSV-2 or HP-PRRSV-2, co-cultured with PBL, and stimulated with either ConA or PMAi served as PRRSV-2-inoculated control. Monocytes treated with rTGFβ1, co-cultured with PBL, then inoculated with either cPRRSV-2 or HP-PRRSV-2 (0 h), and stimulated with inducers (48 h) served as rTGFβ1-treated/PRRSV-2-inoculated control. Monocytes treated with rIFNα, co-cultured with PBL, then inoculated with either cPRRSV-2 or HP-PRRSV-2 (0 h), and stimulated with inducers (48 h) served as rIFNα -treated/PRRSV-2-inoculated control. Monocytes co-cultured with PBL receiving mock Ag plus inducers served as uninoculated control. Cell culture supernatants were collected for real-time PCR. The C_T_ values were obtained and PRRSV-2 ORF7 RNA copy numbers were calculated based on the C_T_ standard curve generated from 10^1^-10^8^ copies of recombinant PRRSV-2 ORF7 plasmids. Data were presented in log 10 scale of copy number/ml. Error bars indicate the SD. Mean differences of PRRSV-2 ORF7 RNA copy numbers among groups at time points were tested by one-way repeated measures ANOVA, followed by Tukey HSD. Different letters indicate significant differences. P<0.05 was set as a statistically significant level.

The direct effect of rTGFβ1 on PRRSV-2 RNA yields was examined, along with its effect on the anti-PRRSV activity of rIFNα. Compared to cPRRSV-2-inoculated monocytes co-cultured with PBL, monocytes treated with rTGFβ1 prior to cPRRSV-2 inoculation and the addition of PBL for co-culture demonstrated significantly higher amount of PRRSV-2 ORF7 RNA copy numbers at 12, 24, 36, 48, and 60 h after inoculation (**Figures 8A, B**). Compared to HP-PRRSV-2-inoculated monocytes co-cultured with PBL, monocytes treated with rTGFβ1 prior to HP-PRRSV-2 inoculation and PBL addition demonstrated significantly higher amount of PRRSV-2 ORF7 RNA copy numbers at 12, 24, 36, 48, and 60 h after inoculation (**Figures 8A, B**).

Compared to cPRRSV-2-inoculated monocytes co-cultured with PBL, monocytes treated with rIFNα prior to cPRRSV-2 inoculation and PBL co-culture demonstrated a significantly lower amount of PRRSV-2 ORF7 RNA copy numbers at 12, 24, 36, 48, and 60 h after inoculation (**Figures 8A, B**). Compared to HP-PRRSV-2-inoculated monocytes co-cultured with PBL, monocytes treated with rIFNα prior to HP-PRRSV-2 inoculation and addition of PBL demonstrated significantly lower amount of PRRSV-2 ORF7 RNA copy numbers at 12, 24, 36, 48, and 60 h after inoculation (**Figures 8A, B**).

Compared to cPRRSV-2-inoculated monocytes co-cultured with PBL, monocytes treated with rTGFβ1, followed by rIFNα prior to cPRRSV-2 inoculation and PBL co-culture showed no alteration of the amount of PRRSV-2 ORF7 RNA copy numbers after inoculation (**Figures 8A, B**). Likewise, compared to HP-PRRSV-2-inoculated monocytes co-cultured with PBL, monocytes treated with rTGFβ1, followed by rIFNα prior to HP-PRRSV-2 inoculation and PBL co-culture did not show alteration of the amount of PRRSV-2 ORF7 RNA copy numbers after inoculation (**Figures 8A, B**). No PRRSV-2 ORF7 RNA was detected in cells treated with mock Ag.

## 4 Discussion

The effects of PRRSV-induced TGFβ1 overexpression on mRNA expressions of transcription factors, type I and II IFNs, MHCs, anti-inflammatory and pro-inflammatory cytokines in PRRSV-2-inoculated cells were investigated in this study. PRRSV-2-induced TGFβ1 overexpression in cells e.g., monocytes, MDMs, and in lungs and lymphoid organs of pigs has been reported (27, 34, 35). At present, the detailed function of PRRSV-2-up-regulated TGFβ1 expression on swine immune protection against PRRSV-2 has not yet been explored.

The phosophorothioate-modified TGFβAS ODN targeting the AUG region of TGFβ1 mRNA significantly reduced TGFβ1 mRNA expression and protein translation (**Figure 2**, **Additional File 3**). In swine immune setting, the AUG region is reported as the most efficient target for gene knockdown of at least cytokines i.e., IFNγ, TGFβ1, and IL-10 (49, 50). Theoretically, phosophorothioate-modified AS ODNs hybridize specifically to target region of mRNA, which enables RNaseH to cleave the hybridized target mRNA. This consequentially results in the degradation of the intact mRNA template for protein translation.

Porcine monocytes are permissive to PRRSV-2 infection in the PBMC population. In the previous research, adherent porcine monocytes in the well-plate had greater than 90% of SWC3a^+^, which is indicative of monocytes (44). Non-adherent cells in PBMCs consisting of PBLs incubated for 2 h has been demonstrated to yield CD3^+^ lymphocytes and with less than 5% of SWC3^+^ cells in the harvested population (26). To indicate T-helper lymphocyte polarization, transcription factors i.e., FOXP3, T-bet, GATA3, and RORγT, together with immune-related genes i.e., TGFβ1, IFNγ, IL4, and IL17, were chosen as representative indicators of Treg, Th1, Th2, and Th17, respectively.

Significantly increased mRNA expressions of FoxP3, GATA3, IL-2, IL-4, IL-10, IL-12p40, IL-17, RORγT, TGFβ1, and T-bet were detected in monocytes co-cultured with PBL and inoculated with either cPRRSV-2 or HP-PRRSV-2. HP-PRRSV-2-inoculated cells significantly had higher mRNA expression levels of Foxp3, IL-10, and TGFβ1 than cPRRSV-2-inoculated monocytes co-cultured with PBLs. Similar levels of mRNA expressions of GATA3, IL-2, IL-12p40, IL-17, RORγT, and T-bet were observed between cPRRSV-2 and HP-PRRSV-2-inoculated monocytes co-cultured with PBLs.

Similar findings have been reported in PRRSV-2-infected PBMCs that expressed high levels of FoxP3 (26), IL-2 (45), IL-10, and TGFβ1 (27). It was recorded that PRRSV N protein (10) and GP5 (21) induce IL-10 production *in vivo* and ex vivo. The increased expression of TGFβ1 in PRRSV-infected MDMs *in vitro*, and in lungs, lymph nodes and tonsils of PRRSV-infected pigs has been reported (34, 35). In addition, Foxp3 and IL-10 mRNA expressions were also elevated in PRRSV-2-infected moDCs (6, 10) and MDMs (7). Also, significant upregulation of GATA3 mRNA expression in PRRSV-infected mononuclear phagocyte cells co-cultured with lymphocytes was recorded (51). In addition, enhanced IL-4 production in PRRSV-2-infected pigs was observed (25). RORγt contributes to the increased IL-17 production. IL-17 was induced in HP-PRRSV-2-infected PAMs by inducing P13K and p38MAPK signaling (52). PRRSV-infected pigs have been recorded with upregulated T-bet expression (53) in lung, tracheobronchial lymph node, and thymus (54). Upregulated IL-12p40 expression in lungs (55) and moDCs (56) was also demonstrated in PRRSV-infected pigs. IL-12p40 expression through the JNK-AP-1 and NF-κB signaling pathways *in vitro* and *in vivo* (55). These findings indicated that immune-related gene expressions associated with Treg, Th1, Th2 and Th17 cells in response to PRRSV-2 inoculation were activated.

In contrast to the upregulation of FoxP3, GATA3, IL-2, IL-4, IL-10, IL-12p40, IL-17, RORγT, TGFβ1, and T-bet mRNA expressions, both cPRRSV-2 and HP-PRRSV-2 downregulated the mRNA expressions of type I and II IFNs (i.e., IFNα and IFNγ), MHCs (i.e., MHC-I, MHC-II), STATs (i.e., STAT1, STAT2 and STAT6) and proinflammatory cytokines (i.e., TNFα) (**Figure 4**, **Additional File 4**). The mRNA expression levels of IFNα, IFNγ, MHC-I, MHC-II, STAT1, STAT2, and TNFα were lower in HP-PRRSV-2-inoculated monocytes and PBL co-culture than in cPRRSV-2-inoculated monocytes and PBL co-culture.

Similar findings have been reported in PRRSV-infected pigs. PRRSV proteins i.e., nsp1α, nsp1β, nsp2, nsp4, nsp11, and N, have been identified as type I IFN antagonists by targeting CREB-binding protein (CBP) (13, 14), NF-κβ signaling (57), NEMO (58), STAT2 and ISG production (59, 60), STAT1 translocation (12), and IRF3 phosphorylation (11), respectively. IFNα and IFNγ transcription levels in PRRSV-infected PAMs were upregulated at 12 and 24 h post-infection, and significantly down-regulated at 36–72 h post-infection (61). Downregulations of type I and II in MDMs (7), monocytes (62), and PBMCs (27) inoculated with PRRSV-2 have been demonstrated. MHC-I and MHC-II expressions were suppressed in PRRSV-infected APCs (6, 56, 63). PRRSV-2 was reported to reduce STAT1, STAT2, and STAT6 expressions in MARC-145 cells and PAMs (64, 65). HP-PRRSV was demonstrated to inhibit TNFα in PAMs. PRRSV’s nsp1 downregulates TNFα production by targeting ERK signaling, NF-κβ, and SP1 promoter activities (21, 66, 67). It has been suggested that PRRSV-induced suppression of innate immunity potentially causes poor adaptive immune responses (68), characterized by attenuated T lymphocyte proliferation, and poor induction of PRRSV-specific IFN-γ-producing cells (69).

TGFβAS1 transfection in monocytes then co-cultured with PBL and inoculated with cPRRSV-2 and HP-PRRSV-2 significantly reduced TGFβ1 mRNA expression (**Figures 3A, C**). Percentage reductions of 41% and 38% were observed on TGFβAS1-transfected/cPRRSV-2-inoculated cells then stimulated with ConA and PMAi, respectively. Also, there were 25% and 27% reductions in TGFβAS1-transfected/HP-PRRSV-2-inoculated cells then stimulated with ConA and PMAi, respectively. ConA and PMAi were used to induce mRNA expressions of TGFβ1 and other immune-related genes of interest. In the presence of these inducers, we clearly demonstrate that PRRSV-2 suppresses mRNA expressions of several immune-related genes in PRRSV-inoculated cells as compared to uninoculated control (45, 49). Unexpectedly, mRNA expressions of IL-4 and IL-10 were significantly reduced with transfection of TGFβAS1 in monocytes co-cultured with PBL and inoculated with cPRRSV-2 and HP-PRRSV-2 (**Figure 4**, **Additional File 4**). The significant reductions of IL-4 and IL-10 mRNA expressions were not detected in the specificity testing of TGFβAS1 in uninoculated monocytes and PBL co-culture (**Table 1**). Approximately, there were 18% and 11% reductions of IL-4 and IL-10 in TGFβAS1-transfected/cPRRSV-2-inoculated cells, respectively. Whereas, TGFβAS1-transfected/HP-PRRSV-2-inoculated cells had approximately 22% and 5% reductions in IL-4 and IL-10 mRNA expressions, respectively. The outcomes of reduced IL-4 and IL-10 expressions in TGFβAS1-transfected/PRRSV-2-inoculated monocytes and PBL co-culture were not clearly understood. IL-10 and TGFβ reportedly promote each other’s gene expression (70). In human monocytes and macrophages, TGFβ induces IL-10 production (71). In murine T-cells, TGFβ suppresses Th2 by inducing T-regs (72). IL-4 primarily drives Th2 polarization. Th1 cells produce IFNγ that inhibits IL-4-mediated Th2 differentiation (73). In pigs, IL-4 expression has been shown to control APC’s inflammatory activities. IL-4 was recorded to induce porcine PAMs to become an alternatively activated M2 macrophages that are characterized by IL-10 production (74). In both human and murine, IL-4 is vital for antibody production and a diagnostic marker for Th2 immune cell response. In contrast, IL-4 is not a porcine B-cell’s stimulatory factor because it impedes IL-6 and antibody secretion, and suppresses antigen-stimulated proliferation of B-cells (75). IL-4 was also demonstrated to suppress the transcription of inflammatory cytokines in porcine macrophages (76). The slightly reduced IL-4 and IL-10 after TGFβ knockdown might be attributed to the upregulation of STAT1/2 and IFNα/γ. This hypothesis needs to be verified by more studies, as no significant differences in T-bet expression were observed in PRRSV-2-inoculated and TGFβAS1-transfected/PRRSV-2-inoculated monocytes and PBL co-culture.

In contrast to the significant reductions of IL-4, IL-10, and TGFβ1 mRNA in response to TGFβAS1 transfection, TGFβAS1-transfected monocytes co-cultured with PBLs and then inoculated with cPRRSV-2 and HP-PRRSV-2 significantly increased IFNα, IFNγ, MHC-I, MHC-II, STAT1, and STAT2 mRNA expressions (**Figure 4**, **Additional File 4**). In addition, mRNA expressions of IL-12p40 were also significantly reduced in TGFβAS1-transfected cells then inoculated with cPRRSV-2. The existing information regarding the immunoregulatory activities of TGFβ in pigs is limited. In murine T-cells, TGFβ inhibits Th1-mediated inflammatory response and cell differentiation by suppressing IFNγ, T-bet, IL-2, and IL-4 but promotes Tregs differentiation (41, 77, 78). In porcine MDMs, TGFβ has been reported to down-regulate CD14, IL-6, MHC-II, and TNFα (37). In murine macrophages, TGFβ has been reported to inhibit TLR2, TLR4, and TLR5 ligand-induced NF-κβ activation, TNFα, CD14, CD40, IL12p40, and MHCII (38, 39). The increased mRNA expressions of immune-related genes in response to TGFβAS1 transfection should be considered alongside the reduced IL-10 expression. IL-10 has been shown to suppress mRNA expression of these immune-related genes (79). In pigs, knocking down IL-10 led to significantly increased mRNA expressions of IFNγ and TNFα, and slightly increased CD80, CD86, IL-1β, and IL-12p40 mRNA expressions (45, 46).

The transfection of monocytes with TGFβAS1, followed by co-culturing with PBLs and subsequent inoculation with cPRRSV-2 and HP-PRRSV-2, resulted in a significant decrease in PRRSV-2 ORF7 RNA copy numbers (**Figure 5**). This reduction was observed from 12 h to 60 h after inoculation. In TGFβAS1-transfected/cPRRSV-2-inoculated cells, the reduction percentages of PRRSV-2 ORF7 RNA copy numbers were approximately 10.8% at 12 h after inoculation and 28.5% at 60 h after inoculation. Similarly, in TGFβAS1-transfected/HP-PRRSV-2-inoculated cells, the reduction percentage was approximately 9.6% at 12 h after inoculation and 24.6% at 60 h after inoculation. It is important to note that the reduction in PRRSV-2 ORF7 RNA copy numbers was not attributed to non-specific binding of TGFβAS1 to PRRSV RNA, as there was no alignment between TGFβAS1 and any ORFs of cPRRSV-2 and HP-PRRSV-2 used in this study. Furthermore, it is worth mentioning that the reduction percentage of PRRSV-2 ORF7 RNA copy numbers and TGFβ1 mRNA expression was higher in TGFβAS1-transfected/cPRRSV-2-inoculated monocytes and PBL co-culture compared to TGFβAS1-transfected/HP-PRRSV-2-inoculated monocytes and PBL co-culture.

In addition to the notable decrease in TGFβ1 mRNA expression, a significant decrease in PRRSV-2 ORF7 RNA copy numbers was observed in TGFβAS1-transfected/cPRRSV-2-inoculated and TGFβAS1-transfected/HP-PRRSV-2-inoculated monocytes and PBL co-culture, accompanied by a significant increase in mRNA expressions of IFNα, IFNγ, MHC-I, MHC-II, STAT1, and STAT2. Previous studies have reported the inhibitory effects of certain immune-related genes, such as IFNα, IFNγ, and TNFα, on PRRSV replication (80–82). To further investigate the role of these immune-related genes in reducing PRRSV-2 ORF7 RNA copy numbers, commercially available rIFNα was utilized. Treatment of monocytes and PBL co-culture with an optimal concentration of rIFNα prior to either cPRRSV-2 or HP-PRRSV-2 inoculation resulted in a significant reduction in PRRSV-2 ORF7 RNA copy numbers (**Figure 7**). Monocytes were susceptible to PRRSV-2 infection in the co-culture system. Knockdown of TGFβ1 in monocytes within the co-culture system led to the upregulation of IFNα, IFNγ, MHC-I, MHC-II, STAT1, and STAT2, which could potentially act as the anti-PRRSV response. These findings suggest that the significant increase in the expressions of these immune-related genes in response to TGFβ1 knockdown may contribute to the reduction of PRRSV-2 ORF7 RNA copy numbers.

rTGFβ1 was employed to provide additional clarification regarding the potential role of PRRSV-up-regulated TGFβ1 expression in supporting PRRSV replication. The treatment of monocytes and PBL co-culture with rTGFβ1 prior to inoculation with either cPRRSV-2 or HP-PRRSV-2 resulted in a significant increase in PRRSV-2 ORF7 RNA copy numbers (**Figure 6**). Additionally, the treatment of cells with rTGFβ1 prior to rIFNα treatment and inoculation with cPRRSV-2 or HP-PRRSV-2 led to a reduction in the antiviral activity of rIFNα (**Figure 8**). These findings demonstrate the positive influence of TGFβ1 on PRRSV replication. Furthermore, these findings suggest a potential strategy employed by PRRSV to enhance virus replication and diminish innate immune defense against the virus through the upregulation of TGFβ1 expression.

## 5 Conclusion

In conclusion, both cPRRSV-2 and HP-PRRSV-2 significantly upregulated the expression of TGFβ1 in the co-culture of monocytes and PBL. The knockdown of TGFβ1 expression by TGFβAS1 significantly enhanced the IFNα/γ, MHC-I/II, and STAT1/2 mRNA expression levels in the monocytes and PBL co-cultures infected with the virus. Additionally, the suppression of TGFβ1 expression by TGFβAS1 contributed to the significant reduction in the yields of PRRSV-2 RNA copy numbers. Conversely, rTGFβ1 and rIFNα sustained and decreased the yields of PRRSV-2 RNA copy numbers, respectively. The results of this study illustrate a plausible strategy employed by PRRSV to suppress the innate immune response, highlighting the immunomodulatory role of PRRSV-induced TGFβ in dampening the innate immune defense against the virus. Furthermore, these findings suggest that the development of future PRRSV vaccines and vaccine adjuvants should consider targeting TGFβ as a potential therapeutic approach.

## Data availability statement

The original contributions presented in the study are included in the article/**Supplementary Material**. Further inquiries can be directed to the corresponding author.

## Ethics statement

The animal study was approved by Animal Care and Use Committee, Maejo University (Approval number MACUC 014S/2562). The study was conducted in accordance with the local legislation and institutional requirements.

## Author contributions

DF: Conceptualization, Data curation, Formal analysis, Investigation, Methodology, Project administration, Validation, Visualization, Writing – original draft, Writing – review & editing. WC: Conceptualization, Funding acquisition, Resources, Supervision, Writing – review & editing.
