# Supplementary material for: Type I and II interferons, transcription factors and major histocompatibility complexes were enhanced by knocking down the PRRSV-induced transforming growth factor beta in monocytes co-cultured with peripheral blood lymphocytes
Source: Front Immunol. 2024 Mar 6;15:1308330. doi: 10.3389/fimmu.2024.1308330 (PMC10950996; doi:10.3389/fimmu.2024.1308330)
Supplement: Supplementary file 1 [file DataSheet_1.docx]

**Additional File 1** Primer details and optimized conditions for all genes presented in this study.

| **Gene** | **Primer sequence (5’ ->3’)** | **Primer**  **location** | **Target sequence** | **Size**  **(bp)** | **Ta^*^**  **(°C)** | **Primer**  **Conc.**  **(nM)** | **E^*^**  **(%)** | **r^2^** | **Tm^*^**  **(^o^C)** |
| --- | --- | --- | --- | --- | --- | --- | --- | --- | --- |
| FoxP3 | F: AATGGTCAAAGCTGACTTTCCTG  R: TGTTCCAGAGACTGCACCAC | 622-641  819-800 | NM_001128438.1 | 198 | 55 | 500 | 95 | 0.999 | 86 |
| GATA3 | F: AACCACGTCCCGTCCTACTA  R: GGTGGATGGACGTCTTGGAG | 305-324  503-484 | NM_001044567.1 | 199 | 60 | 300 | 102 | 0.996 | 88 |
| IFNα^a^ | F: AGCCTCCTGCACCAGTTCTG  R: TCACAGCCAGGATGGAGTCC | 346-365  469-450 | NM_214393.1 | 124 | 60 | 300 | 97 | 0.996 | 86 |
| IFNγ^b^ | F: TGGTAGCTCTGGGAAACTGAATG  R: GGCTTTGCGCTGGATCTG | 342-364  420-403 | NM_213948 | 79 | 60 | 400 | 102 | 1.000 | 78 |
| IL-2 | F: CAAACGGTGCACCTACTTCA  R: CCTGCTTGGGCATGTAAAAT | 53-72  208-227 | NM_213861.1 | 156 | 60 | 300 | 92 | 0.991 | 79 |
| IL-4 | F: TCTCACCTCCCAACTGATCC  R: AAGGTTTCCTTCTCCGTCGT | 29-48  229-210 | NM_214123.1 | 201 | 60 | 300 | 100 | 0.999 | 81 |
| IL-6^e^ | F: CTGGCAGAAAACAACCTGAACC  R: GATTCTCATCAAGCAGGTCTCC | 316-337  409-387 | NM_214399.1 | 94 | 55 | 300 | 100 | 0.994 | 78 |
| IL-10^b^ | F: CGGCGCTGTCATCAATTTCTG  R: CCCCTCTCTTGGAGCTTGCTA | 430-450  518-498 | NM_214041 | 89 | 60 | 200 | 96 | 0.999 | 82 |
| IL-12p40 | F: GACCTCGGGGAGTATAAGAAGT  R: TTGTGAACAGCTTCCAGCAC | 588-609  694-675 | NM_214013.1 | 107 | 60 | 500 | 100 | 0.990 | 84 |
| IL-17 | F: TCATGATCCCACAAAGTCCA  R: AGTCCATGGTGAGGTGAAGC | 130-149  275-256 | NM_001005729.1 | 146 | 55 | 500 | 99 | 0.990 | 84 |
| MHC-I | F: GAGAAGGAGGGGCAGGACTA  R: AACATGCTCTGGAGGGTGTG | 251-270  390-371 | NM_001097431.1 | 140 | 60 | 300 | 94 | 0.990 | 84 |
| MHC-II | F: TCTGTGCGGTGACAGATTTC  R: ATCTCCTCGCTGGAGATTCA | 494-513  654-635 | NM_001113694.2 | 161 | 55 | 500 | 94 | 1.000 | 82 |
| RORγT | F: ACGTGGTGGAGTTCGCTAAG  R: CTGACCAGCACCACTTCCAT | 1929-1948  2031-2012 | XM_021089853.1 | 103 | 55 | 500 | 93 | 0.998 | 85 |
| RPL32^c^ | F: CGGAAGTTTCTGGTACACAATGTAA  R: TGGAAGAGACGTTGTGAGCAA | 249-273  342-322 | NM_001001636.1 | 94 | 55 | 400 | 99 | 0.993 | 79 |
| STAT1 | F: GTGATCTCCAACGTCAGCCA  R: AAACTGCCAACTCAGCACCT | 1369-1388  1518-1499 | NM_213769.1 | 150 | 55 | 300 | 102 | 0.993 | 84 |
| STAT2 | F: GCACTGGGCAACGATGATTC  R: TGGAATGGCCTGAATGTCCC | 223-242  375-356 | NM_213889.1 | 153 | 60 | 400 | 97 | 0.999 | 82 |
| STAT6 | F: AAGTTCATGGCTGAGGTGGG  R: CACTGCCAAAAGGTGAAGCC | 1520-1539  1698-1679 | NM_001197306.1 | 179 | 55 | 300 | 98 | 0.997 | 84 |
| T-bet | F: ACTCTCCTCTCCTCCCCAAC  R: GCTGAGGGCAGAAATGTAGG | 1144-1163  1315-1296 | NM_001315722.1 | 172 | 60 | 300 | 100 | 0.990 | 86 |
| TGFβ1^b^ | F: TACGCCAAGGAGGTCACCC  R: CAGCTCTGCCCGAGAGAGC | 1215-1233  1370-1352 | NM_214015.1 | 156 | 60 | 400 | 101 | 0.991 | 84 |
| TNFα | F: AGCCTCTTCTCCTTCCTCCTG  R: GAGACGATGATCTGAGTCCTTGG | 283-303  427-405 | NM_214022 | 145 | 60 | 300 | 93 | 0.994 | 84 |
| YWHAZ^a^ | F: ATTGGGTCTGGCCCTTAACT  R: GCGTGCTGTCTTTGTATGACTC | 961-980  1106-1085 | XM_001927228.4 | 146 | 58 | 300 | 91 | 0.998 | 80 |

FOXP3, forkhead box P3; IFN, interferon; IL, interleukin; MHC, major histocompatibility complex; RORγT, retinoid-related orphan receptor gamma T; RPL32, 60S ribosomal protein L32; STAT, signal transducer and activator of transcription; T-bet, t-box expressed in T cells; TGFβ1, transforming growth factor beta; TNFα, tumor necrosis factor alpha; YWHAZ, tyrosine 3-monooxygenase/tryptophan 5-monooxygenase activation protein, zeta

^*^Ta, Annealing temperature; E, Efficiency from serial dilutions of reference cDNA; Tm, Melting temperature

^a^Fossum, C., Hjertner, B., Ahlberg, V., Charerntantanakul, W., Mcintosh, K., Fuxler, L., Balagunaseelan, N., Wallgren, P., and Lovgren Bengtsson, K. (2014). Early inflammatory response to the saponin adjuvant Matrix-M in the pig. *Vet Immunol Immunopathol* 158**,** 53-61

^b^ Wikstrom, F.H., Fossum, C., Fuxler, L., Kruse, R., and Lovgren, T. (2011). Cytokine induction by immunostimulatory DNA in porcine PBMC is impaired by a hairpin forming sequence motif from the genome of Porcine Circovirus type 2 (PCV2). *Vet Immunol Immunopathol* 139**,** 156-166

^c^ Duvigneau JC, Hartl RT, Groiss S, Gemeiner M. (2005). Quantitative simultaneous multiplex real-time PCR for the detection of porcine cytokines. *Journal of Immunological Methods*. 306(1-2),16-27

Primers without superscript letters were originally designed in this study.
