# Supplementary material for: Type I and II interferons, transcription factors and major histocompatibility complexes were enhanced by knocking down the PRRSV-induced transforming growth factor beta in monocytes co-cultured with peripheral blood lymphocytes
Source: Front Immunol. 2024 Mar 6;15:1308330. doi: 10.3389/fimmu.2024.1308330 (PMC10950996; doi:10.3389/fimmu.2024.1308330)
Supplement: Supplementary file 2 [file DataSheet_2.docx]

**
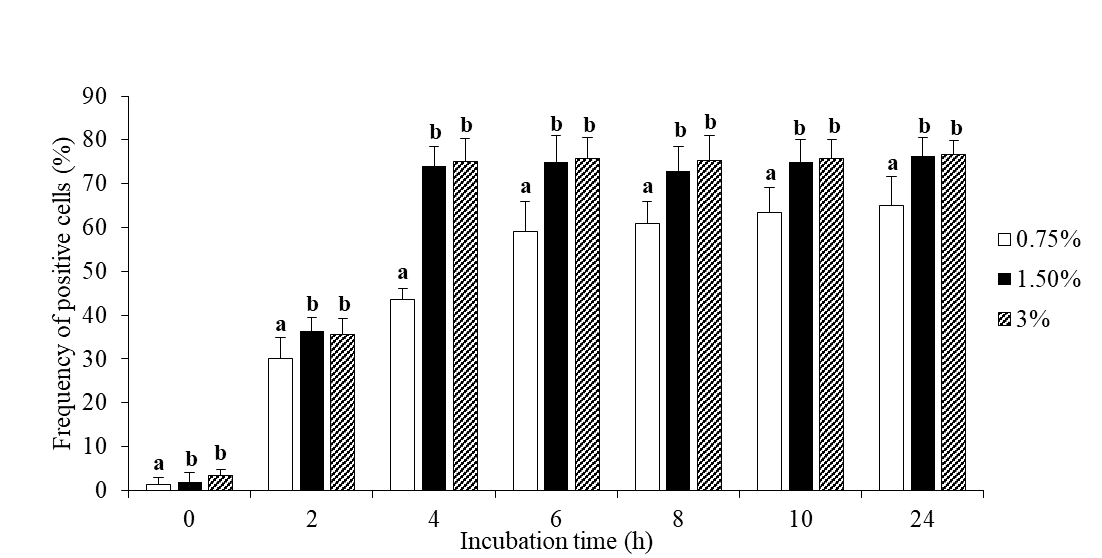
**

**Additional File 2** Monocyte uptake of fluorescent-labeled siRNA complexed with different concentrations (0.75%, 1.5%, 3% v/v) of transfection reagent. Mean differences of percentages of fluoresced cells among groups at time points were tested by one-way repeated measures ANOVA, followed by Tukey HSD test. Different letters indicate significant difference. P<0.05 was set as a statistically significant level.
