# Supplementary material for: Type I and II interferons, transcription factors and major histocompatibility complexes were enhanced by knocking down the PRRSV-induced transforming growth factor beta in monocytes co-cultured with peripheral blood lymphocytes
Source: Front Immunol. 2024 Mar 6;15:1308330. doi: 10.3389/fimmu.2024.1308330 (PMC10950996; doi:10.3389/fimmu.2024.1308330)
Supplement: Supplementary file 3 [file DataSheet_3.docx]

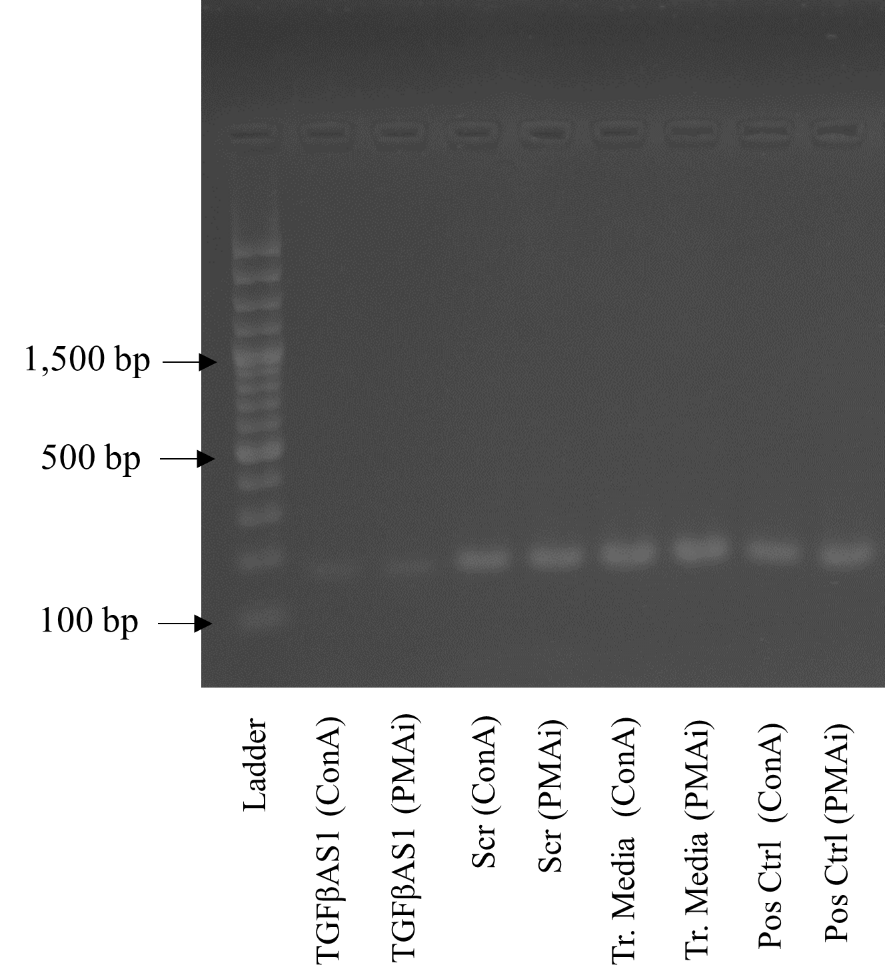


**Additional file 3** Band intensities indicate the quality of TGFβ1 knockdown (refer to Figure 2A and 2C). Monocytes were transfected with TGFβAS1 or Scr, added with PBL, then stimulated with inducers (either ConA or PMAi). Monocytes transfected with transfection media (Tr.media) alone, added with PBL, and finally stimulated with inducers served as Tr.media control. Untransfected monocytes, added with PBL, and finally stimulated with inducers served as positive control (Pos Ctrl).
