# Supplementary material for: Type I and II interferons, transcription factors and major histocompatibility complexes were enhanced by knocking down the PRRSV-induced transforming growth factor beta in monocytes co-cultured with peripheral blood lymphocytes
Source: Front Immunol. 2024 Mar 6;15:1308330. doi: 10.3389/fimmu.2024.1308330 (PMC10950996; doi:10.3389/fimmu.2024.1308330)
Supplement: Supplementary file 4 [file DataSheet_4.docx]

**Additional file 4**  Effects of TGFβAS1 transfection on expression levels of immune-related genes in monocytes and PBL co-culture, inoculated with either cPRRSV-2 or HP-PRRSV-2 and stimulated with either ConA or PMAi.

| **Gene** | **cPRRSV-2** | **HP-PRRSV-2** | **Tr.Media+ cPRRSV-2** | **Tr.Media+HP-PRRSV-2** | **TGFβAS1+ cPRRSV-2** | **TGFβAS1+ HP-PRRSV-2** | **Mock Ag** | **Pos Ctrl** |  |
| --- | --- | --- | --- | --- | --- | --- | --- | --- | --- |
|  |  |  |  |  |  |  |  |  |  |
| **FoxP3** | 2.6 ± 0.3^a^ | 3.1 ± 0.2^b^ | 2.6 ± 0.2^a^ | 3.1 ± 0.2^b^ | 2.5 ± 0.4^a^ | 2.9 ± 0.2^a,b^ | 2.1 ± 0.1^c^ | 2.1 ± 0.2^c^ |  |
| **GATA3** | 3.3 ± 0.3^a^ | 3.3 ± 0.1^a^ | 3.4 ± 0.2^a^ | 3.4 ± 0.1^a^ | 3.0 ± 0.4^a^ | 3.1 ± 0.2^a^ | 2.7 ± 0.3^b^ | 2.7 ± 0.3^b^ |  |
| **IFNα** | -0.2 ± 0.4^a^ | -0.8 ± 0.4^b^ | -0.1 ± 0.4^a^ | -0.7 ± 0.3^b^ | 1.8 ± 0.4^c^ | 1.3 ± 0.2^d^ | 3.8 ± 0.4^e^ | 3.7 ± 0.4^e^ |  |
| **IFNγ** | 0.7 ± 0.3^a^ | -0.2 ± 0.3^b^ | 0.8 ± 0.3^a^ | -0.1 ± 0.3^b^ | 3.2 ± 0.3^c^ | 2.5 ± 0.4^d^ | 4.8 ± 0.3^e^ | 4.9 ± 0.4^e^ |  |
| **IL-2** | 3.9 ± 0.3^a^ | 4.1 ± 0.1^a^ | 4.0 ± 0.1^a^ | 3.9 ± 0.4^a^ | 3.8 ± 0.4^a^ | 3.9 ± 0.2^a^ | 3.0 ± 0.2^b^ | 3.1 ± 0.1^b^ |  |
| **IL-4** | 2.8 ± 0.3^a^ | 3.1± 0.2^b^ | 2.9 ± 0.2^a,b^ | 3.1 ± 0.2^b^ | 2.3 ± 0.2^c^ | 2.4 ± 0.2^c^ | 2.5 ± 0.2^c^ | 2.5 ± 0.2^c^ |  |
| **IL-6** | 2.9 ± 0.4^a^ | 3.4 ± 0.3^a^ | 3.0 ± 0.3^a^ | 3.3 ± 0.3^a^ | 2.6 ± 0.4^a^ | 3.0 ± 0.3^a^ | 3.0 ± 0.3^a^ | 2.9 ± 0.3^a^ |  |
| **IL-10** | 5.4 ± 0.2^a^ | 6.0 ± 0.1^b^ | 5.4 ± 0.1^a^ | 6.1 ± 0.1^b^ | 4.8 ± 0.3^c^ | 5.7 ± 0.1^d^ | 4.5 ± 0.1^e^ | 4.5 ± 0.1^e^ |  |
| **IL-12p40** | 3.9 ± 0.3^a^ | 4.1 ± 0.2^a^ | 4.0 ± 0.3^a^ | 4.2 ± 0.3^a,b^ | 4.6 ± 0.3^b^ | 4.3 ± 0.3^a,b^ | 3.3 ± 0.3^c^ | 3.3 ± 0.3^c^ |  |
| **IL-17** | 3.5 ± 0.3^a,b^ | 3.8 ± 0.2^a,b^ | 3.4 ± 0.3^a,c^ | 3.9 ± 0.3^b^ | 3.5 ± 0.2^a,b^ | 3.9 ± 0.2^b^ | 2.9 ± 0.4^c,d^ | 2.9 ± 0.4^d^ |  |
| **MHC-I** | 0.3 ± 0.2^a^ | -0.4 ± 0.2^b^ | 0.2 ± 0.3^a^ | -0.3 ± 0.2^b^ | 0.8 ± 0.4^c^ | 0.2 ± 0.2^c^ | 2.3 ± 0.4^d^ | 2.2 ± 0.2^d^ |  |
| **MHC-II** | -1.6 ± 0.2^a^ | -2.4 ± 0.1^b,c^ | -2.0 ± 0.4^a,c^ | -2.5 ± 0.2^b^ | 0.9 ± 0.4^d^ | 0.3 ± 0.4^e^ | 2.2 ± 0.3^f^ | 2.3 ± 0.2^f^ |  |
| **RORγT** | 4.2 ± 0.2^a^ | 4.1 ± 0.1^a^ | 4.2 ± 0.2^a^ | 4.2 ± 0.1^a^ | 4.2 ± 0.2^a^ | 4.1 ± 0.4^a^ | 2.7 ± 0.4^b^ | 2.7 ± 0.4^b^ |  |
| **STAT1** | 1.8 ± 0.2^a^ | 1.5 ± 0.1^b^ | 1.8 ± 0.2^a^ | 1.4 ± 0.1^b^ | 2.3 ± 0.2^c^ | 1.8 ± 0.1^a^ | 2.3 ± 0.4^c^ | 2.1 ± 0.3^c^ |  |
| **STAT2** | -0.3 ± 0.3^a^ | -0.9 ± 0.2^b^ | -0.3 ± 0.1^a^ | -1.0 ± 0.1^b^ | 1.7 ± 0.4^c^ | 1.2 ± 0.2^c^ | 2.6 ± 0.2^d^ | 2.5 ± 0.1^d^ |  |
| **STAT6** | 1.4 ± 0.2^a^ | 1.6 ± 0.1^a^ | 1.4 ± 0.2^a^ | 1.5 ± 0.2^a^ | 1.6 ± 0.2^a^ | 1.5 ± 0.1^a^ | 2.2 ± 0.1^b^ | 2.1± 0.1^b^ |  |
| **T-bet** | 3.8 ± 0.2^a^ | 3.8 ± 0.2^a^ | 4.0 ± 0.3^a^ | 3.9 ± 0.2^a^ | 3.9 ± 0.2^a^ | 3.8 ± 0.2^a^ | 3.4 ± 0.2^b^ | 3.4 ± 0.1^b^ |  |
| **TNFα** | 0.1 ± 0.3^a,c^ | -0.5 ± 0.2^b^ | 0.0 ± 0.3^a,c^ | -0.6 ± 0.2^b^ | 0.3 ± 0.4^c^ | -0.2 ± 0.2^a,b^ | 4.3 ± 0.4^d^ | 4.4 ± 0.4^d^ |  |

Monocytes were transfected with TGFβAS1, then reintroduced with PBL and inoculated with either cPRRSV-2 or HP-PRRSV-2, and finally stimulated with inducers; either ConA (for IFNα, IFNγ, IL-6, IL-17, IL-12p40, RORγT, Stat1, T-bet, TNFα) or PMAi (for MHC-I, MHC-II, IL-2, IL-4, FoxP3, Stat2, Stat6, GATA3, IL-10). Untransfected monocytes and PBL co-culture them inoculated with cPRRSV-2 or HP-PRRSV-2 and stimulated with inducer served as PRRSV-2-inoculated control. Monocytes treated with transfection media (Tr.media), then added with PBL and inoculated with cPRRSV-2 or HP-PRRSV-2, then stimulated with inducer served as PRRSV-2-inoculated/Tr. media control. Monocytes and PBL co-culture inoculated with mock Ag and stimulated with inducers served as mock control. Untreated co-culture of monocyte and PBL receiving culture media in the presence or absence of inducer served as positive and negative controls, respectively. Error bars indicate the SD. Mean differences of immune gene expressions among groups were tested by one-way ANOVA, followed by Tukey HSD test. Different letters indicate significant difference. P<0.05 was set as a statistically significant level.
